# Cardiac structural predictors of ventricular tachycardia…looking beyond ejection fraction and ventricular scar

**DOI:** 10.1186/1532-429X-17-S1-P190

**Published:** 2015-02-03

**Authors:** Navdeep Tandon, John A Apostolis, Nairmeen A Haller, Vinayak A Hegde

**Affiliations:** Cardiology, Akron General Medical Center, Copley, OH USA; Cardiology, North East Ohio Medical University, Rootstown, OH USA

## Background

Each year, about 424,000 adults experience sudden cardiac death (SCD) in the United States. Assessment for cardiac structural anatomy is vital in this population. While left ventricular ejection fraction (LVEF) is an established predictor of ventricular tachycardia (VT), it is known that VT occurs in nonischemic patients, with normal LVEF. Contribution of ventricular scar is also under investigation. Our study aims to assess the role of newer cardiac magnetic resonance (CMR) based parameters in this population.

## Methods

We performed a retrospective chart review of 49 patients who underwent CMR exams between July 2010-2012 (23 without, and 26 with sustained or nonsustained VT/ aborted SCD). Patients with ventricular tachycardia were subgrouped as patients with (11), and without (15) ventricular scar. Patients with hypertrophic cardiomyopathy were excluded from the study. All patients with VT were proven to have nonocclusive coronary artery disease by coronary angiography prior to CMR study. CMR based parameters including myocardial mass (MM), atrial scar, ventricular scar, ventricular volumes, and LVEF were recorded on these patients. Chi square test and t test were used to analyze differences between VT and control groups. Subgroup analyses included comparing the VT subgroup without ventricular scars against the controls.

## Results

Our analysis suggested a higher incidence of atrial scar in patients with ventricular tachycardia 54% vs 22% in controls (c^2^ (1, N=49) =5.3, p = 0.037). This difference was not observed when the subgroup of VT pts without ventricular scar was compared against controls. Also, there was a trend towards a lower average MM in the VT group (146 gm ± 47) as compared to the control group (189 gm ± 102), although this difference did not reach statistical significance (t (30.32) =-1.83, p= 0.07). End diastolic, end systolic, and stroke volumes were not statistically different between the study groups.

## Conclusions

The cause of VT remains obscure in 33% of patients despite imaging with echocardiography and CMR. Our study highlights the potential for using alternate CMR derived parameters such as atrial scarring as predictors of VT/SCD. Although a difference in myocardial mass between groups was not observed, the trend towards a lower MM in patients with VT is promising. Repeating the study prospectively, with more patients may explore the role of these novel parameters further.

## Funding

None.

